# Cerebral venous sinus thrombosis and aneurysm in a patient with double heterozygous beta-thalassemia major: A case report

**DOI:** 10.1097/MD.0000000000026082

**Published:** 2021-05-28

**Authors:** Rui Gu, Yao Xiong, Li Li, Xiaoling Zhao, Yan Liu

**Affiliations:** Department of Neurology, The Third People's Hospital of Chengdu, The Affiliated Hospital of Southwest Jiaotong University, Chengdu, Sichuan Province, China.

**Keywords:** aneurysm, case report, cerebral venous sinus thrombosis, thalassemiaThe authors have no funding to disclose

## Abstract

**Rationale:**

Thalassemia is an inherited disease associated with thromboembolic events (TEE) and cerebral artery disease. Here, we report a patient with beta-thalassemia presenting with intracerebral hemorrhage due to cerebral venous sinus thrombosis (CVST), and intracranial aneurysms were found after examination. We believe that it is very rare for this patient to have two kinds of cerebrovascular diseases.

**Patients’concern:**

A 25-year-old woman suffered from headache for nine days. She had a history of thalassemia and splenectomy nine years prior.

**Diagnosis:**

Intracranial hemorrhage, Cerebral venous sinus thrombosis, Intracranial aneurysm and double heterozygous beta-thalassemia major.

**Interventions:**

The patient was treated with low-molecular-weight heparin sodium injection (4100IU sc q12 h) and then switched to warfarin after four days of overlap with low-molecular-weight heparin sodium injection. Oral hydroxyurea was prescribed before discharged from the hospital.

**Outcomes:**

The patient's headache was relieved significantly within 48 h, and re-examination of CT showed that the hemorrhage was completely absorbed one week later.

**Lessons:**

CVST and intracranial aneurysms are associated with the pathological mechanism of thalassemia, and patients with beta-thalassemia should be monitored and educated for long-term prevention, especially those with risk factors.

## 1 Introduction

Thalassemia is an inherited disease with a high prevalence in Mediterranean^[1]^ and South China. It is estimated that the gene carrier rate in this region is between five and 70%.^[2]^ Thalassemia is categorized as alpha or beta-thalassemia, depending on the type of globin chain synthesis disorder. According to the clinical characteristics it can be divided into three subtypes: thalassemia major (TM), thalassemia intermedia (TI) and thalassemia minor. Thalassemia affects at least thousands of children each year, and has become a global public health problem.^[3]^

Thalassemia has been reported to be associated with thromboembolic events (TEE) and cerebral artery disease. However, to our knowledge, there are no reports of cerebral venous sinus thrombosis (CVST) in patients with thalassemia. The patient reported in this study had two intracranial aneurysms. For this reason, we regard it as a rare case that deserves to be reported.

## 2 Case presentation

A 25-year-old woman in Sichuan (southwest China) was admitted to the hospital because of a sudden headache for nine days. She had a history of thalassemia and splenectomy nine years prior. Physical examination revealed that the patient was pale and without any neurological deficits. In this case report, we did not provide an experimental intervention, so there was no need for ethical review. Written informed consent was obtained from the patient.

**Figure 1:**
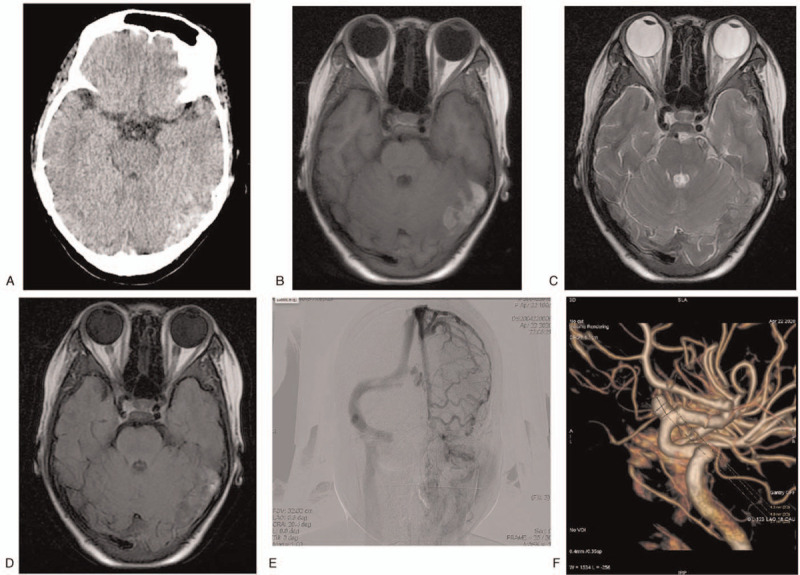
(A). Brain CT showed little hemorrhage in the left occipital lobe. (B). Brain MRI(T1WI) showed high signal lesion located in the left lateral transverse sinus and sigmoid sinus area of the left occipital region. (C). Brain MRI (T2WI) showed low signal lesion. (D). Brain MRI (FLAIR) showed mixed signal lesion. (E). DSA showed left transverse sinus and sigmoid sinus occlusion, and filling defect of right transverse sinus. (F). DSA showed two aneurysms located in the C6 segment of the right ICA with the size of 4.9^∗^5.7 mm and 2.1^∗^1.3 mm.

Laboratory examinations showed the following indicators: white blood cells, 11.41∗10^9/L hemoglobin, 58 g/L; hematocrit, 20.6%; and platelets, 702∗10^9/L. Hemoglobin electrophoresis showed hemoglobin A 0.00% (96.8%–97.8%), hemoglobin F 50% (0–2.0%), and hemoglobin A2 3.6% (2.2%–3.2%). The serum ferritin level was 1733ng/ml. Brain computed tomography (CT) showed a small hemorrhage in the left occipital lobe (Fig. 1A). Therefore, the diagnosis of intracranial hemorrhage is clear. However, the patient was young and had significant blood abnormalities; therefore, secondary intracranial hemorrhage was highly suspected. We performed brain magnetic resonance imaging (MRI) and cerebral digital subtraction angiography (DSA). MRI showed an irregular lesion located in the left lateral transverse sinus and sigmoid sinus area of the left occipital region with T1WI high signal, T2WI low signal, and FLAIR mixed signal (Fig. 1B–D). DSA showed left transverse sinus and sigmoid sinus occlusion, filling defect of right transverse sinus, two aneurysms located in the C6 segment of the right internal carotid artery(ICA) with the size of 4.9∗5.7 mm and 2.1∗1.3 mm (Fig. 1E,F). For blood abnormalities, we performed a genetic test to screen for the cause of anemia. The gene mutation of globin in the peripheral blood samples of the patient was analyzed and identified using polymerase chain reaction-reverse dot blot (PCR-RDB). The results showed that there was double heterozygosity for IVS-II-654 (HBB:c.316_197C > T) and Hb E (HBB:c.79G > A). The diagnosis was intracranial hemorrhage, cerebral venous sinus thrombosis, intracranial aneurysm and double heterozygous beta-thalassemia major.

The patient was treated with low-molecular-weight heparin sodium injection (4100IU sc q12 h), and her headache was relieved significantly within 48 h. One week later, a re-examination of the CT scan showed that the hemorrhage was completely absorbed. Her treatment was switched to warfarin after four days of overlap with low-molecular-weight heparin sodium injection, and she started taking hydroxyurea, and she was discharged from the hospital.

The patient is in stable condition and is being followed up regularly by the hematology department.

## 3 Discussion

Thalassemia is associated with a high risk of developing TEE.^[4]^ The major underlying mechanisms are platelet activation, abnormal erythrocyte surface activation, endothelial cell activation, and iron overload.^[1]^ A clinical study^[4]^ involving 8860 patients indicated that TI had a higher risk of TEE than TM, including deep venous thrombosis (DVT) (32%), stroke (18%), portal vein thrombosis (16%), pulmonary embolism (13%), and superficial thrombophlebitis (4.7%). More venous thrombosis occurred in the TI, and more arterial thrombosis occurred in the TM.^[4]^ Most patients with TI who had TEE were splenectomized (94%), had an average hemoglobin level <90 g/L (68%), and had not received regular blood transfusion (n=2/3). In addition, TEE rates were significantly lower in patients receiving aspirin. A systematic review^[5]^ showed that cerebral TEE occurred in 1.13% of patients with beta-thalassemia, and splenectomy was a risk factor. Another study also confirmed that splenectomy, elevated platelet counts (≥500∗10^9/L), and serum ferritin levels ≥1000 ng/ml are risk factors for developing TEE in thalassemia.^[6,7]^ One study analyzed the time interval between TEE and splenectomy. The median time was eight years, which indicated that TEE in patients with splenectomy is a manifestation of a chronic underlying process that further emphasizes the need for a long-term treatment modality for prevention.^[7]^ However, none of the studies mentioned thrombosis of the cerebral venous system. To the best of our knowledge, there have been no reports of CVST associated with thalassemia. This patient was a double heterozygous beta-thalassemia case with splenectomy nine years ago and did not undergo regular blood transfusion with a much higher platelet count and serum ferritin level. The patient's clinical characteristics met almost all the risk factors for TEE.

The literature on intracranial aneurysms in thalassemia is rare, with controversial conclusions. A study on magnetic resonance angiography (MRA) in splenectomized patients with TI indicated that 17.2% had aneurysms and 27.6% had arterial stenosis. ICA was the most commonly involved artery (75%).^[8]^ Several case reports of subarachnoid hemorrhage in beta-thalassemia indirectly support that beta-thalassemia has an increased risk of aneurysm.^[9]^ It is generally believed that the pathophysiological mechanisms are oxidative vessel injury and endothelial dysfunction associated with iron overload.^[10]^ However, a controlled study showed that there was no difference in the incidence of aneurysms or size between beta-thalassemia and control.^[11]^ A three-year MRI and MRA report recorded nine patients with beta-thalassemia harboring asymptomatic intracranial aneurysms.^[12]^ The results showed that aneurysms did not change in size or shape. However, it is worth noting that all the patients recorded in the study had relatively small aneurysms at baseline (2–5 mm) with a very low PHASES aneurysm risk score. In the case of our patient, her PHASE score is zero but she had two aneurysms with the diameter of the larger one is above 5 mm. Therefore, we believe that the aneurysms should be treated. Considering the low risk of rupture in a short time and the high risk of stent thrombosis if we administer interventional therapy to patients immediately before the hypercoagulation was corrected, we decided to wait until hydroxyurea and warfarin work.

## 4 Conclusion

Herein, we report a case of double heterozygous beta-thalassemia major with CVST and intracranial aneurysms. We are convinced that these two types of cerebral vascular disease occurring in one person are associated with thalassemia. More studies on the relationship between cerebral vascular disease and thalassemia are needed. Patients with beta-thalassemia should be monitored for TEE and educated for long-term prevention, especially those with risk factors.

## Author contributions

**Conceptualization:** rui gu.

**Data curation:** rui gu, Yao Xiong, Li Li, Xiaoling Zhao, Yan Liu.

**Methodology:** rui gu.

**Writing – original draft:** rui gu, Yao Xiong.

**Writing – review & editing:** rui gu.
